# Study of the lithium diffusion properties and high rate performance of TiNb_6_O_17_ as an anode in lithium secondary battery

**DOI:** 10.1038/s41598-017-16711-9

**Published:** 2017-11-30

**Authors:** Yong-Seok Lee, Kwang-Sun Ryu

**Affiliations:** Department of Chemistry, University of Ulsan, Ulsan, 680-749 Korea

## Abstract

TiNb_6_O_17_ and TiNb_2_O_7_ were synthesized using a solid-state method. The techniques were used to assess the electrochemical performance and lithium diffusion kinetics of TiNb_6_O_17_ related to the unit cell volume with TiNb_2_O_7_. The charge-discharge curves and cyclic voltammetry revealed TiNb_6_O_17_ to have a similar redox potential to TiNb_2_O_7_ as well as a high discharge capacity. The rate performance of TiNb_6_O_17_ was measured using a rate capability test. SSCV and EIS showed that TiNb_6_O_17_ had higher lithium diffusion coefficients during the charging. From GITT, the lithium diffusion coefficients at the phase transition region showed the largest increase from TiNb_2_O_7_ to TiNb_6_O_17_.

## Introduction

Lithium secondary batteries have been studied for large scale energy devices, such as electric vehicles (EVs) and energy storage systems (ESSs), requiring high energy density and superior rate performance. The development of anode materials has been investigated due to importance of the charge rate and good reversibility for lithium secondary batteries. Commercial anode materials for batteries, such as graphite, have high capacities (370mAh/g). On the other hand, the active material has some problems, such as irreversible capacity loss, due to solid electrolyte interface (SEI) layer and lithium dendrite formation due to the low working voltage window at 0.8 V^1^. In particular, lithium dendrite formation leads to the safety hazard of lithium secondary batteries and the unsuitability of the active materials for batteries^1–3^. The Si based materials such as SiO_2_ showed also high capacity but could not be used to high volume expansion^4^. In contrast, titanium-based anode materials allow lithium batteries to avoid SEI and lithium dendrite formation due to their safe working voltage area using the Ti^4+^/Ti^3+^ redox reaction (~1.5 V vs. Li/Li^+^)^2^. Typically, Li_4_Ti_5_O_12_ has been studied because of its working voltage area and zero strain properties, resulting in good rate performance due to its strong Ti-O covalent bond^3^. Despite this, the material has a low theoretical capacity (175mAh/g) and is unsuitable for large-scale devices.

**Figure 1:**
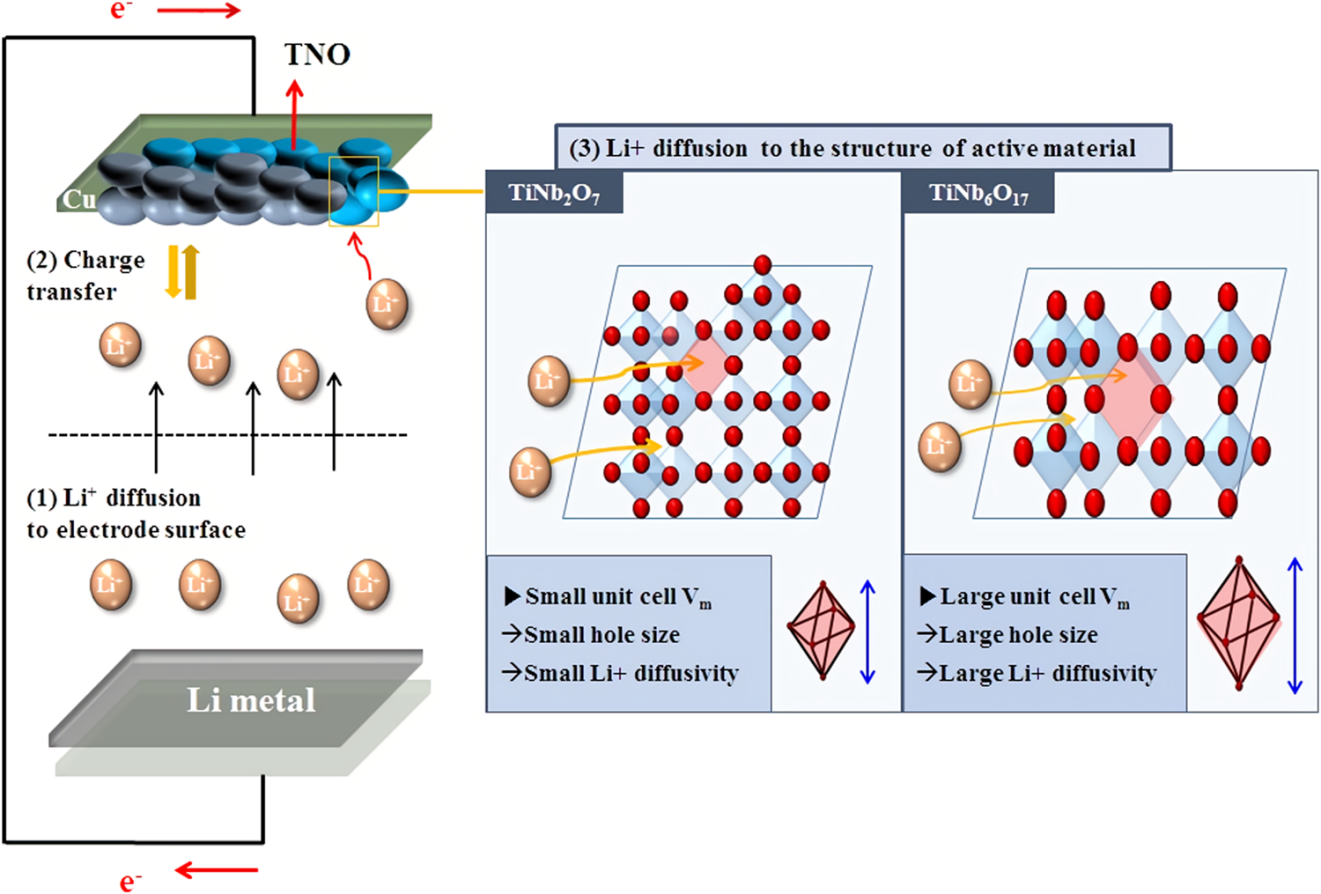
Schematic diagram of the kinetic mechanism of lithium diffusion in Li secondary batteries and phenomena about the unit cell size.

Recently, titanium niobium oxide (TNO) materials, such as TiNb_2_O_7_ and Ti_2_Nb_10_O_29_, have been introduced as promising titanium-based anode materials owing to their nontoxic, good rate performance, low volume change, stable working voltage window (1–2.5 V), and high theoretical capacity (387~390mAh/g). The capacities of TNO materials are influenced by many redox reactions, such as one Ti (Ti^3+^/Ti^4+^) and two Nb reactions (Nb^3+^/Nb^4+^ and Nb^4+^/Nb^5+^)^5,6^. On the other hand, they have lower capacity and reversibility than their theoretical capacities due to the low electric conductivity and lithium diffusion properties into the structure called Wesley-Roth 2D structure^7–9^. To solve these problems, many studies have been conducted to achieve TNO materials with high reversible capacity and improved rate performance, such as doping with other metals (Ru, Mo, etc.) to achieve high ionic conductivity and electrical conductivity and controlling the particle shape and size^1–3,6–12^. Chunfu Lin *et al*. examined TiNb_6_O_17_, which is a new TNO material. The material is composed a large number of Nb ions and has a higher theoretical capacity (397 mA/g) than TiNb_2_O_7_ and Ti_2_Nb_10_O_29_. Moreover, the material has the same Wisely-Roth structure (monoclinic) but larger lattice parameters and unit cell volume than TiNb_2_O_7_ and Ti_2_Nb_10_O_29_ (1122.541 Å vs. 803.21 Å, 1118.512 Å) due to the larger number of Nb^5+^ ions with a larger size (0.64 Å) than that of Ti^4+^ ions (0.605 Å)^13–15^. This causes a more open lithium insertion/insertion site and improved rate performance; the schema of this theory is listed in Fig. 1. The material showed a higher discharge capacity and better lithium diffusion coefficients by charge-discharge, rate capability, and slow scan cyclic voltammetry (SSCV) than Ti_2_Nb_10_O_29_ in Chunfu’s study^13^.

Therefore, this study examined the accurate lithium diffusion kinetics and electrochemical performance of TiNb_6_O_17_ compared to TiNb_2_O_7_ which has the smallest unit cell volume among the TNO materials and can clearly be compared with TiNb_6_O_17_. The materials were synthesized using a solid-state method. For electrochemical analysis, the charge-discharge curves and rate capability tests were conducted to determine their electrochemical performance. To examine the lithium diffusion kinetics, SSCV, electrochemical impedance spectroscopy (EIS), and a galvanostatic intermittent titration technique (GITT) were used. As a result, TiNb_6_O_17_ showed higher discharge capacity (284mAh/g vs. 264mAh/g) and better rate performance than TiNb_2_O_7_ (82mAh/g vs. 20mAh/g at 30 C). In addition, TiNb_6_O_17_ showed higher lithium diffusion coefficients than TiNb_2_O_7_ (mean value 10^–12^ S^2^/m vs. 10^–13^ S^2^/m).

## Experimental

### Synthesis of the active materials and characterization

TiNb_2_O_7_ and TiNb_6_O_17_ were synthesized by a solid-state reaction method using TiO_2_ (99.9%, Rare Metallic) and Nb_2_O_5_ (99.99%, Sigma-Aldrich) powders as the starting materials. TiO_2_ and Nb_2_O_5_ were mixed by ball milling at a stoichiometric molar ratio for 4 h at 300 rpm. The mixed powder was pressed into pellets and calcined in air 1300 °C for 12 h (5 °C/min). The morphology and Ti and Nb content in the two TNO materials were observed by field-emission scanning electron microscopy (FE-SEM, Jeol JSM6500F) and energy dispersion spectroscopy (EDS) attached to FE-SEM. The crystalline structures of the materials were analyzed by X-ray powder diffraction (XRD, Rigaku, Ultima4) was conducted using Ka1 radiation at 45KV/40 mA in the range, 10–100° (2θ). Fourier-transform infrared spectroscopy (FT-IR, Shimadzu IR AFFInity-1S) and X-ray photoelectron spectroscopy (XPS, ThermoFisher K-alpha) were used to examine the chemical bonding and oxidation state of the TNO materials, respectively.

### Coin cell assembly and electrochemical analysis

The composition of the TNO anodes was a mixture of active material (TiNb_2_O_7_ or TiNb_6_O_17_, 70 wt. %), conducting agent (Super-P, 20 wt. %), and polyvinylidene fluoride binder (PVdF 5130, 10 wt. %). The materials were mixed by ball-milling in 1-methyl-2-pyrrolidinone (NMP) until a viscous slurry formed and cast on Cu foil. The electrochemical properties were tested in CR2032-type coin cells. The cells were assembled with a TNO electrode as the working electrode and lithium metal as the counter electrode separated by a membrane with polypropylene in an Ar-filled glove box. The electrolyte was 1 M LiPF_6_ dissolved in a mixture of ethylene carbonate (EC) and dimethyl carbonate (DMC) with a volume ratio 1:2. Cyclic voltammetry (CV) was conducted using a battery cycler (Won A tech, WBCS3000) at a scan rate of 0.1mVs^−1^ and ranging from 0.05–0.3 mVs^−1^ from 3.0 to 1.0 V (versus Li/Li^+^). Galvanostatic charge-discharge tests were performed using the battery cycles at 0.1 C (38.7mAg^−1^ of TiNb_2_O_7_ and 39.7mAg^−1^ of TiNb_6_O_17_) from 3.0 to 1.0 V. The rate capabilities were conducted over the voltage range of 3.0–1.0 V with a current density range 1.0 C to 30 C at room temperature. EIS was carried out by applying an AC signal of 5 mV amplitude over the frequency range from 100KHz to 10mHz using an electrochemical analyzer (NeoSience, SP-300). GITT was tested at a current density of 0.1 C over the voltage range of 3.0–1.0 V using the electrochemical analyzer. The procedure of GITT consisted of galvanostatic charge pulses for each duration time (15 min), followed by a relaxation time (30 min).

## Results and Discussion

### Characterization

**Figure 2:**
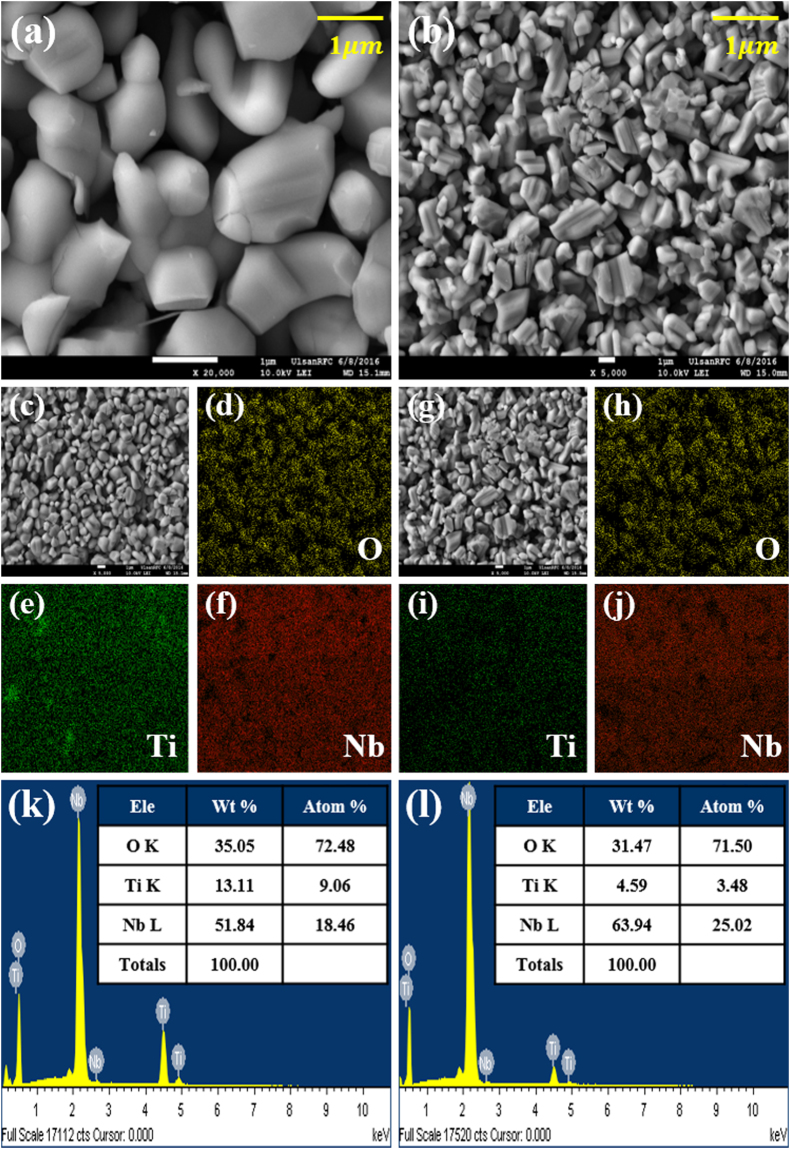
(**a**) SEM images of TiNb_2_O_7_ and (**b**) TiNb_6_O_17_ (magnification ×20,000), (**c**) SEM images of TiNb_2_O_7_ (magnification ×5,000), (**d**)~(**f**) EDS mapping images of oxygen (yellow), titanium (green), and niobium (red), (**g**) SEM images of TiNb_6_O_17_ (magnification ×5,000), and (**h**)~(**i**) EDS mapping images of oxygen, titanium, and niobium, and (**k**) the results of EDS analysis of TiNb_2_O_7_ and (l) TiNb_6_O17.

Figure 2(a),(b) shows SEM images (magnification ×20,000) of TiNb_2_O_7_ and TiNb_6_O_17_. A comparison of the particle size and morphology was not accurate due to irregular particle formation by solid state synthesis. On the other hand, the morphologies of the two materials were similar in principle. The mean particle size of the two samples was approximately 1–3 μm. Figure 2(c)~(f) and (g)~(j) present SEM images of (c) TiNb_2_O_7_ and (g) TiNb_6_O_17_ (magnification ×5,000) and EDS mapping images of (d) oxygen, (e) titanium, and (f) niobium in TiNb_2_O_7_ and (h) oxygen, (i) titanium, and (j) niobium in TiN_6_O_17_. The calculated atomic percentages of the two materials by EDS are presented in Fig. 2(k) TiNb_2_O_7_ and (l) TiNb_6_O_17_. The mapping images of Ti and Nb of the two materials exhibited similar dispersion. On the other hand, the images of Ti in TiNb_2_O_7_ and TiNb_6_O_17_ showed different dispersion and brightness. Ti in TiNb_6_O_17_ was darker than that of TiNb_2_O_7_. The brightness means that the Ti content in TiNb_6_O_17_ is lower than that of TiNb_2_O_7_. These results correspond to atomic percentages of Ti and Nb in the two materials. The atomic percentage ratio of Nb and Ti in TiNb_2_O_7_ was 1:2 (Ti:Nb = 9.06:18.46), whereas the Nb: Ti ratio in TiNb_6_O_17_ was approximately 1:7 (Ti:Nb = 3.48:25.02). These results show that the molar ratio of Nb and Ti is different in the two TNO materials.

**Figure 3:**
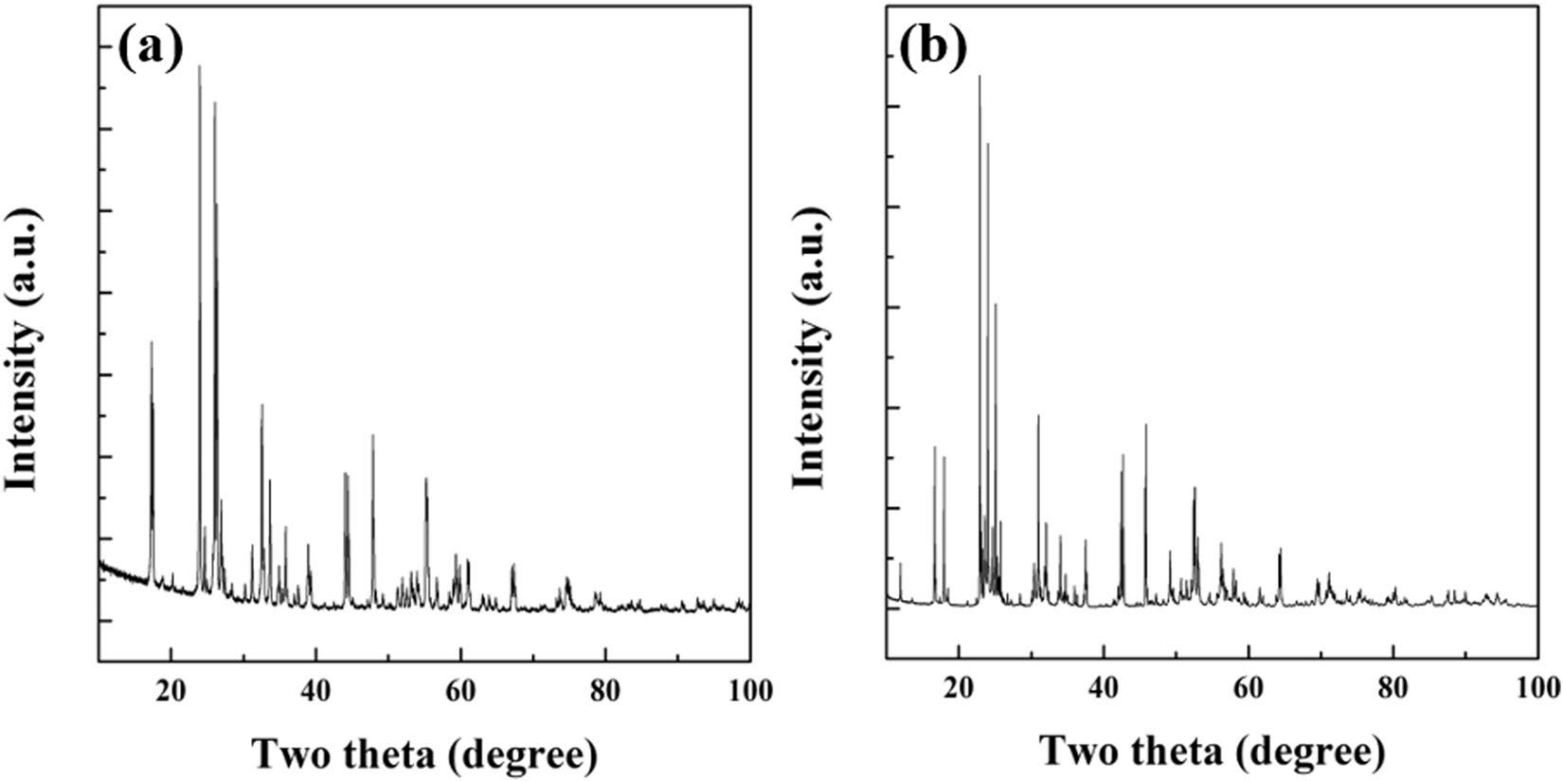
XRD patterns of (**a**) TiNb_2_O_7_ and (**b**) TiNb_6_O_17_.

Figure 3 (a) TiNb_2_O_7_ and (b) TiNb_6_O_17_ present XRD patterns of the two TNO materials. The pattern of TiNb_2_O_7_ was well indexed to the calculated patterns according to the monoclinic symmetry of TiNb_2_O_7_ with the monoclinic ReO_3_ shear structure with the space group C2/m (JCPDS card No. 70–2009);^6^ however, there have been no studies of TiNb_6_O_17_. Therefore, there is no calculated structural data for TiNb_6_O_17_. On the other hand, the XRD patterns of Ti_2_Nb_10_O_29_ have been reported in many studies, which is similar to that of TiNb_6_O_17_^13^. Therefore, TiNb_6_O_17_ has a similar crystal structure to Ti_2_Nb_2_O_29_, which is a Wadsley-Roth shear structure with an A2/m space group. Compared to the XRD patterns of calculated Ti_2_Nb_10_O_29_ and TiNb_6_O_17_ synthesized in this study, most peak positions and intensities were in good agreement except for two main peak intensities, which coincides with the XRD patterns reported by Chunfu Lin. The powder XRD patterns of TiNb6O17 was refined with the fullprof software and the rietveld parameters are a = 15.48089 Å, b = 3.81501 Å, c = 20.62921 Å, α&γ = 90°, β = 113.106°, and *V* *=* 1218.356 Å^3^. The calculated rietveld refinement parameters of TiNb_6_O_17_ is well matched the with the crystalline parameters of Ti_2_Nb_10_O_29_^13^.

**Figure 4:**
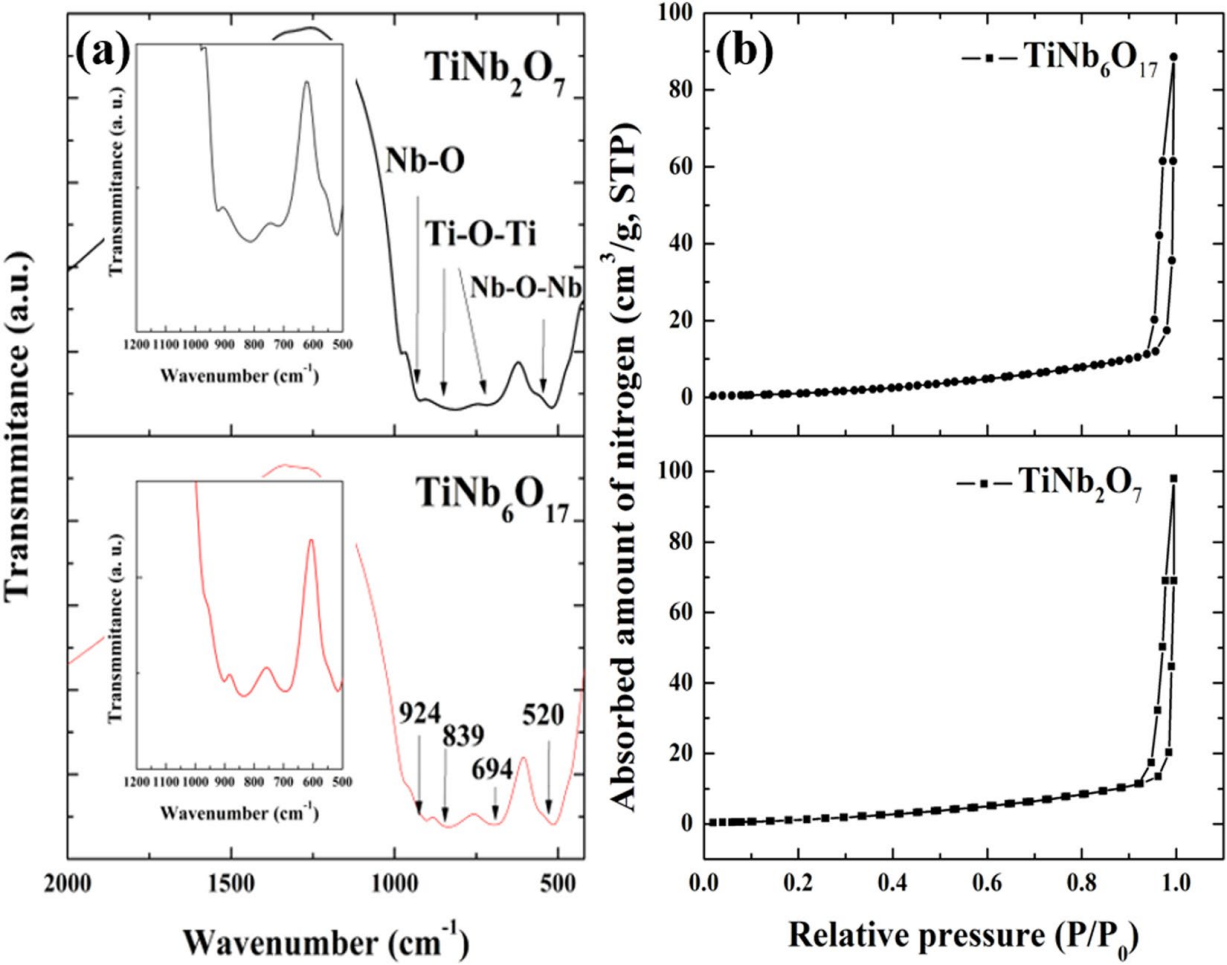
FT-IR spectra in Fig. (**a**) and nitrogen adsorption-desorption isotherm in Fig. (**b**) of two TNO materials.

FT-IR spectroscopy was conducted to characterize the Ti-O and Nb-O bond of TiNb_2_O_7_ and TiNb_6_O_17_. Figure 4(a) presents the FT-IR spectra of two samples. The peaks at 924 cm^−1^ and 520 cm^−1^ correspond to the stretching vibrations of the Nb-O bonds and Nb-O-Nb bridging bonds and the stretching vibration of at 694 cm^−1^ and 839 cm^−1^ are Ti-O-Ti bonds^12^. The BET specific surface area and volume of the TNO materials were studied by nitrogen adsorption techniques; Fig. 4(b) shows the corresponding isotherm. The specific surface area of TiNb_2_O_7_ and TiNb_6_O_17_ is 2.66 m^2^/g and 2.36 m2/g; the mean pore volume of the materials is 0.11 cm^3^/g and 0.10 cm^3^/g respectively. As the measurement was conducted by using standard multi point BET, the specific surface area of two materials is almost same. The results are corresponded to the SEM images showing similar particle size of two materials. Therefore, the surface area of the electrodes made by two TNO materials is also same and have not an effect on the electrochemical analysis such as lithium diffusion analysis.

**Figure 5:**
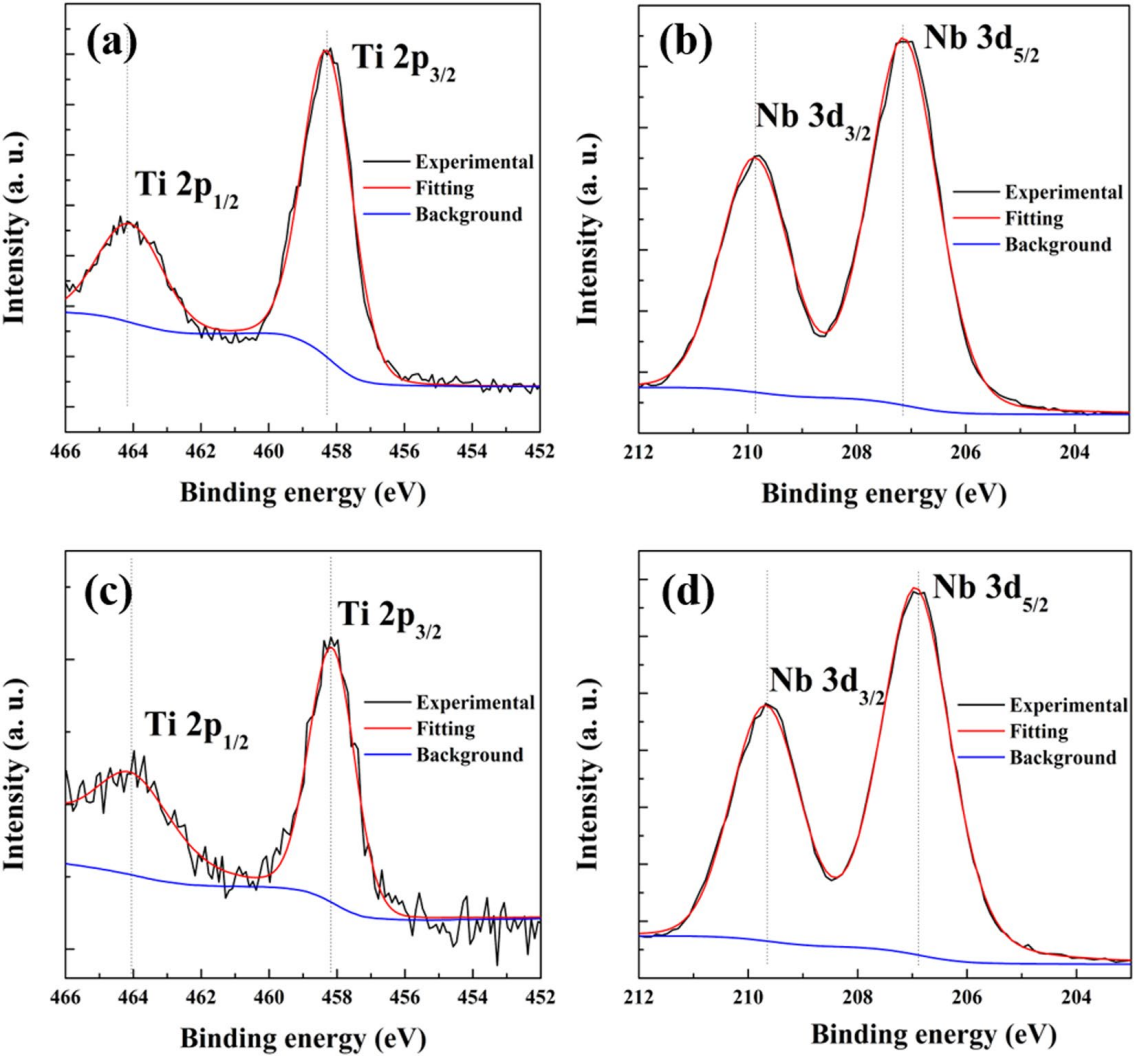
XPS spectra of (**a**) Ti and (**b**) Nb element in TiNb_2_O_7_, (**c**) Ti and (**d**) Nb element in TiNb_6_O_17_.

XPS was used to analyze the chemical oxidation state of Ti and Nb in the samples, as shown in Fig. 5. Figure 5(a) TiNb_2_O_7_ and (c) TiNb_6_O_17_ showed Ti 2p_1/2_ and 2p_3/2_ peaks at 464.18 eV & 458.38 eV (TiNb_2_O_7_), and 464.18 eV & 458.18 eV (TiNb_6_O_17_), respectively. These binding energies were similar and corresponded to the binding energies of Ti^4+^ in TiO_2_^3,5,6,8^. The noise of the Ti spectra was attributed to the smaller content than Nb. In particular, the spectra of Ti in TiNb_6_O_17_ showed more noise than that of TiNb_6_O_17_. This may be because TiNb_6_O_17_ is composed of a lower Ti content than TiNb_2_O_7_. These results match the results of EDS analysis and the mapping images. Figure 5(b) TiNb_2_O_7_ and (d) TiNb_6_O_17_ present the spectra of Nb^5+^ in Nb_2_O_5_.The Nb 3d_3/2_ and Nb 3d_5/2_ peaks were located at (b) 209.88 & 207.18 and (c) 209.68 & 206.98. These values agree with the binding energies of Nb^5+^ in Nb_2_O_5_^3,5,10^. Therefore, FT-IR spectroscopy and XPS shows that the two TNO materials are composed with Ti^4+^ in TiO_2_ and Nb^5+^ in Nb_2_O_5_.

### Electrochemical analysis

**Figure 6:**
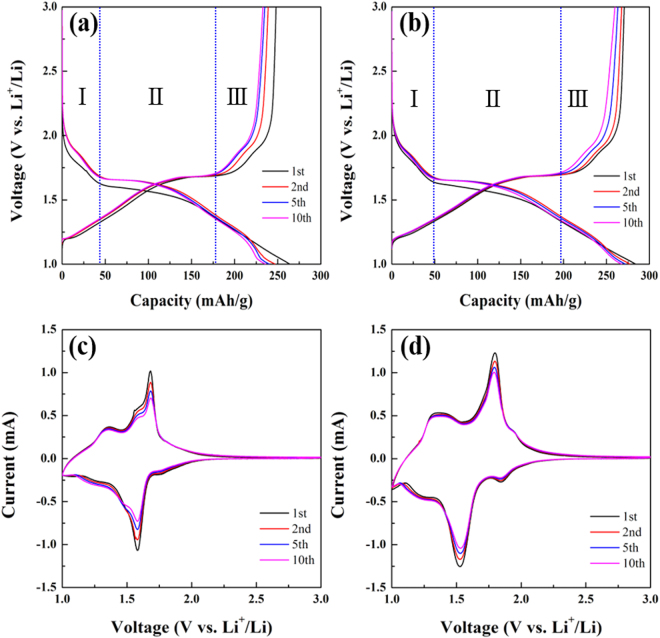
(**a**) Charge/discharge curves of TiNb_2_O_7_ and (**b**) TiNb_6_O_17_anodes at 0.1 C, (**c**) cyclic voltammetry of TiNb_2_O_7_ and (**d**) TIiNb_6_O_17_anodes in the potential window of 1.0–3.0 V at scan rate of 0.1 mVs^−1^.

Figure 6 (a),(b) presents the charge and discharge curves of TiNb_2_O_7_ and TiNb_6_O_17_ at a current density of 0.1 C (38.7 mAg^−1^ and 39.7 mAg^−1^) over the voltage range of 3.0–1.0 V. The curves of the two TNO anodes showed three plateau regions. The regions 1 and 3 are the solid-solution region^6,9^. These regions mean the redox reaction of Ti^4+^ ↔ Ti^5+^ and Nb^3+^ ↔ Nb^4+^, respectively. Region 2 is a two-phase reaction, which means the reaction of Nb^4+^ ↔ Nb^5+^ ^3,5–13^. Compared to the initial discharge capacities, TiNb_6_O_17_ exhibited a larger discharge capacity (284 mAhg^−1^) than that of TiNb_2_O_7_ (264 mAhg^−1^). In addition, the irreversibility of TiNb_6_O_17_ was smaller than TiNb_2_O_7_ particularly from the 1^st^ to 2^nd^ cycles. CV of TiNb_2_O_7_ and TiNb_6_O_17_ was conducted at a scan rate of 0.1mVs^−1^ from 3.0 V to 1.0 V and from 3.0 and 1.0 V for 10 cycles. As shown in Fig. 6(c) TiNb_2_O_7_ and (d) TiNb_6_O_17_, both curves Fig. 6(a) TiNb_2_O_7_ and curve (b) TiNb_6_O_17_ showed three current peaks at the oxidation and reduction state, respectively. Each peak is expressed in the curves (C_p_ and A_p_ mean the cathodic peaks and anodic peaks). Although the reduction peaks were C_p1_ (Ti^4+^ → Ti^3+^), C_p2_ (Nb^5+^ → Nb^4+^), and C_p3_ (Nb^4+^ → Nb^3+^), A_p1_, A_p2_, and A_p3_ mean the oxidation reaction of Nb^3+^ → Nb^4+^, Nb^4+^ → Nb^5+^, and Ti^3+^ → Ti^4+^ ^10,15^. These potential regions of the current peaks were matched with the plateau regions in charge and discharge curves. These results show that the reaction mechanisms of the two TNO materials are the same. In addition, the reaction of Nb^4+^ ↔ Nb^5+^, which is corresponded to two-phase regions in the charge and discharge curves, showed the highest current peak area and is regarded as the main reaction. Compared to the CV curves of TiNb_2_O_7_ and TiNb_6_O_17_, TiNb_6_O_17_ exhibits higher reactivity and reversibility from the peak area at all cycles. In addition, the decrease in the peak intensity during the cycle, particularly A_p2_ and C_p1_, suggests that the reversibility of TiNb_6_O_17_ is better than TiNb_2_O_7_. This is in agreement with the results of the charge and discharge tests.

**Figure 7:**
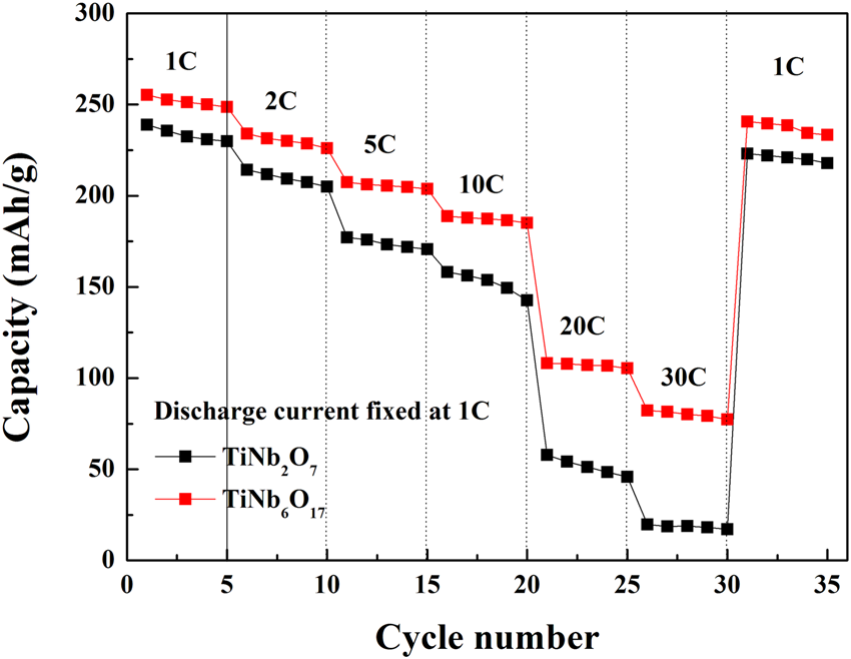
Capacity retention of TiNb_2_O_7_ and TiNb_6_O_17_ anodes at various scan rates (1 C, 2 C, 5 C, 10 C, 20 C, and 30 C); the discharge rate was fixed at 1 C.

To understand the electrochemical performance of the lithium diffusion properties of TiNb_2_O_7_ and TiNb_6_O_17_, the rate capabilities were performed at various C-rates from 1 C to 30 C (discharge rate was fixed at 1 C). Figure 7 presents the rate performance of the two TNO materials. A comparison with the average capacities for the 5^th^cycle at each C-rate revealed TiNb_6_O_17_ to have charge capacities of 252, 230, 206, 187, 107, and 80 mAhg^−1^ at 1 C, 2 C, 5 C, 10 C, 20 C, and 30 C, respectively. These values are larger than that of TiNb_2_O_7_ (234, 210, 174, 152, 52, and 19 mAhg^−1^). In particular, the difference in the charge capacities at a high rate (20 C and 30 C) was distinct. When calculating the ratio of the average charge capacity, 30 C/1 C, the ratio was 8.12% for TiNb_2_O_7_ and 31.7% for TiNb_6_O_17_, which suggests that TiNb_6_O_17_ has better rate properties than TiNb_2_O_7_^13^. In addition, a comparison of the cycling retention at 5 C to 30 C revealed TiNb_6_O_17_ to have better cycling properties, whereas TiNb_2_O_7_ exhibited a rapid decrease in capacity. This means the better electrochemical reversibility of the TiNb_6_O_17_. These studies including the results of the charge and discharge tests and CV indicated that lithium ion transport of TiNb_6_O_17_ is faster than the rate of TiNb_2_O_7_ due to the larger theoretical capacity and better lithium diffusion kinetics by larger lithium site.

**Figure 8:**
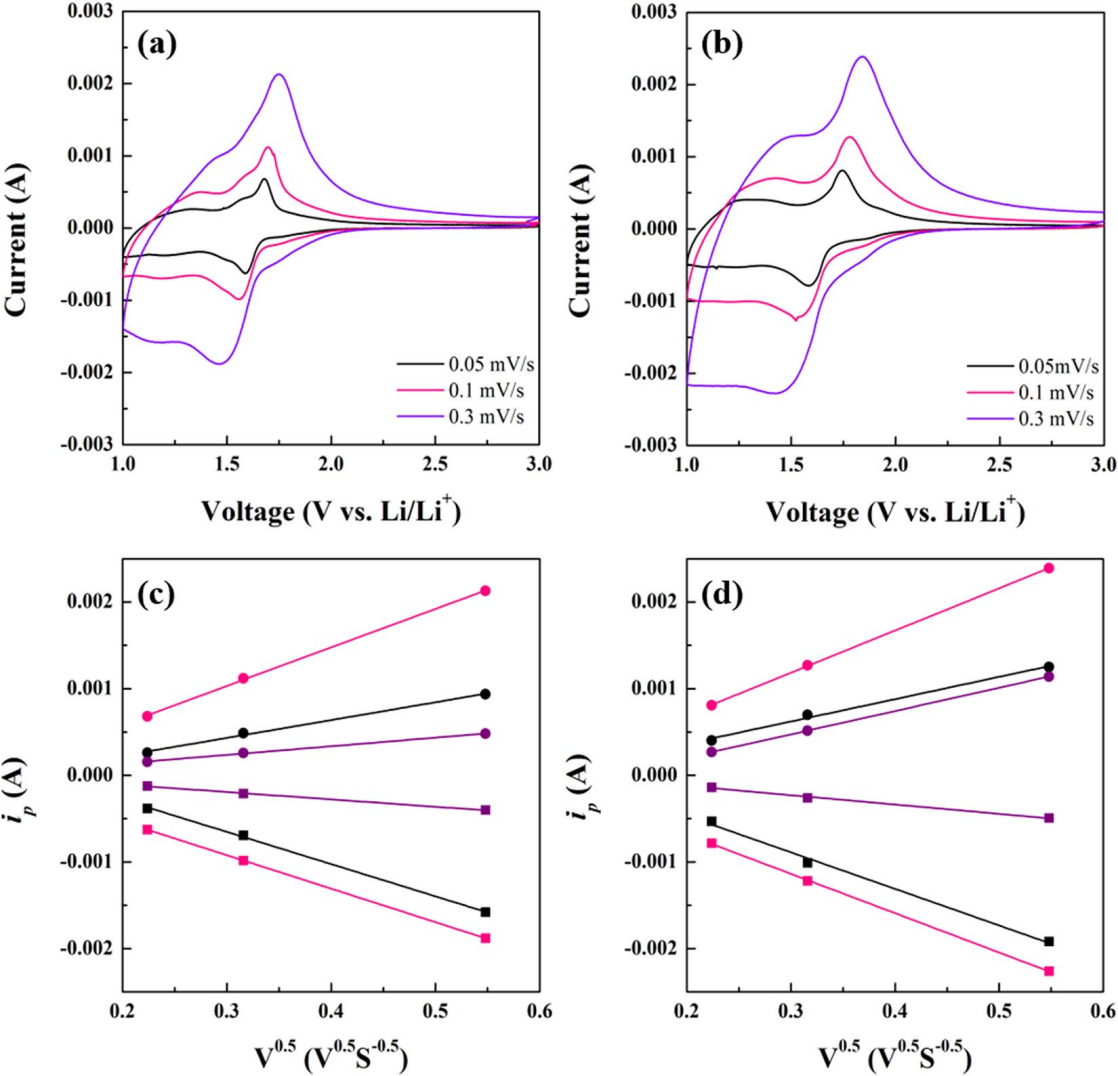
Cyclic voltammetry with various scan rate of (**a**) TiNb_2_O_7_ and (**b**) TiNb_6_O_17_, linear relationship between the peak current of the cathodic/anodic reaction and the square root of the sweep rate (**c**) TiNb_2_O_7_ and (**d**) TiNb_6_O_17_ (●: anodic, ■: cathodic and linear: linear fitting).

Figure 8 presents the CV data of (a) TiNb_2_O_7_ and (b) TiNb_6_O_17_ at various scan rates in the range, 0.05–0.3 mVs^−1^. CV at various scan rates is usually used to study the oxidation and reduction properties in electrochemical reactions and obtain the apparent chemical diffusion coefficient of Li-ions^16–20^. With increasing scan rate, the anodic peaks move to a low potential and the cathodic peaks move to a high potential due to the increasing polarization. In addition, the peak intensities of anodic and cathodic reaction increase with increasing scan rate. The peak current density (*I*_p_) revealed a linear relationship with the square root of the scan rate (*v* ^−0.5^), which is expected for a diffusion-controlled process in Fig. 8(c) TiNb_2_O_7_ and (d) TiNb_6_O_17_^20–22^. Each color means the linearity of three anodic and cathodic peaks (Black: A_p1_ and C_p1_, Pink: A_p2_ and C_p2_, and Purple: A_p3_ and C_p3_). The relationship and chemical diffusion coefficient can be determined from the Randles-Sevcik equation (Eq. 1)^16,17,23^:

1 $$I{\rm{p}}=0.4463{{\rm{n}}}^{3/2}{{\rm{F}}}^{3/2}{{\rm{C}}}_{{\rm{Li}}}+{{\rm{SR}}}^{-1}{{\rm{T}}}^{-1}{{{\rm{D}}}_{({{\rm{Li}}}^{+})}}^{1/2}{v}^{1/2}$$

where n is the charge transfer number; F is Faraday’s constant; C_Li_^+^ is the Li-ion concentration in TiNb_2_O_7_ and TiNb_6_O_17_; S is the surface area per weight of active materials; R is the gas constant; and T is the absolute temperature (K). ${{\rm{D}}}_{{{\rm{Li}}}^{+}}$ is the Li-ion diffusion coefficient, and *v* is the scan rate. In this study, ${{\rm{D}}}_{{{\rm{Li}}}^{+}}$ around three anodic and three cathodic peaks in Fig. 6(c) and (d) was calculated using the above equation. Table 1 lists the calculated ${{\rm{D}}}_{{{\rm{Li}}}^{+}}$. As the results, TiNb_2_O_7_ showed the ${{\rm{D}}}_{{{\rm{Li}}}^{+}}$ value 10^−14^ cm^2^/s which is similar to the diffusion coefficient in the previous study (for phase transition region)^24^. Compared to ${{\rm{D}}}_{{{\rm{Li}}}^{+}}$ at the anodic peaks, ${{\rm{D}}}_{{{\rm{Li}}}^{+}}$ of TiNb_6_O_17_ was 20 times (A_p1_), 12 times (A_p2_), and 38 times (A_p3_) higher than that of TiNb_2_O_7_. ${{\rm{D}}}_{{{\rm{Li}}}^{+}}$ of the peaks A_p1_ (Nb^4+^→ Nb^5+^) and A_p3_ (Ti^3+^→ Ti^4+^) of TiNb_6_O_17_ was particularly high. Although the gap of ${{\rm{D}}}_{{{\rm{Li}}}^{+}}$ at the A_p2_ (Nb^4+^→ Nb^5+^) between TiNb_2_O_7_ and TiNb_6_O_17_ was smaller than those of A_p1_ and A_p3_, the difference was apparent. In the case of ${{\rm{D}}}_{{{\rm{Li}}}^{+}}$ at cathodic peaks, the values of TiNb_6_O_17_ were 5 times (C_p1_, Nb^4+^→ Nb^3+^), 15 times (C_p2_, Nb^5+^→ Nb^4+^), and 14 times (C_p3_, Ti^4+^→ Ti^3+^) higher than those of TiNb_2_O_7_. A comparison of the gap of D between TiNb_6_O_17_ and TiNb_2_O_7_ at the anodic peaks revealed the difference in the D_Li_^+^ values at the cathodic peaks to be low except for C_p2_. On the other hand, the D_Li_^+^ of TiNb_6_O_17_ at A_p2_ and C_p2_ meaning two phase transition in TNO materials were clearly higher than that of TiNb_2_O_7_(12 and 15 times). In addition, the anodic and cathodic reaction of the TNO anodes means the de-lithiation and lithiation process during oxidation and reduction, respectively. Therefore, the lithium diffusion properties of TiNb_6_O_17_ were better than those of TiNb_2_O_7_. The reason is that TiNb_6_O_17_ has a larger unit cell volume and more open Li-ion sites than TiNb_2_O_7_. The advanced crystal structure of TiNb_6_O_17_ leads to a larger size and number of Li-ion transport paths in the crystal structure, facilitating Li-ion transport during the de-lithiation and lithiation processes^10,12,18^.

**Table 1:** Calculated D_Li_^+^ values of (a) TiNb_2_O_7_ and (b) TiNb_6_O_17_ anodes from the CV results.

TiNb_2_O_7_	Anodic peak			Cathodic peak		
**D**_**Li**_^**+**^ **(cm** ^**2**^ **s** ^**−1**^ **)**	A	B	C	A	B	C
	5.69 × 10^−15^	3.01 × 10^−14^	1.60 × 10^−15^	1.16 × 10^−14^	2.33 × 10^−14^	1.08 × 10^−15^
TiNb_6_O_17_	Anodic peak			Cathodic peak		
**D**_**Li**_^**+**^ **(cm** ^**2**^ **s** ^**−1**^ **)**	A	B	C	A	B	C
	1.12 × 10^−13^	3.72 × 10^−13^	6.13 × 10^−14^	5.35 × 10^−14^	3.43 × 10^−13^	1.56 × 10^−14^

**Figure 9:**
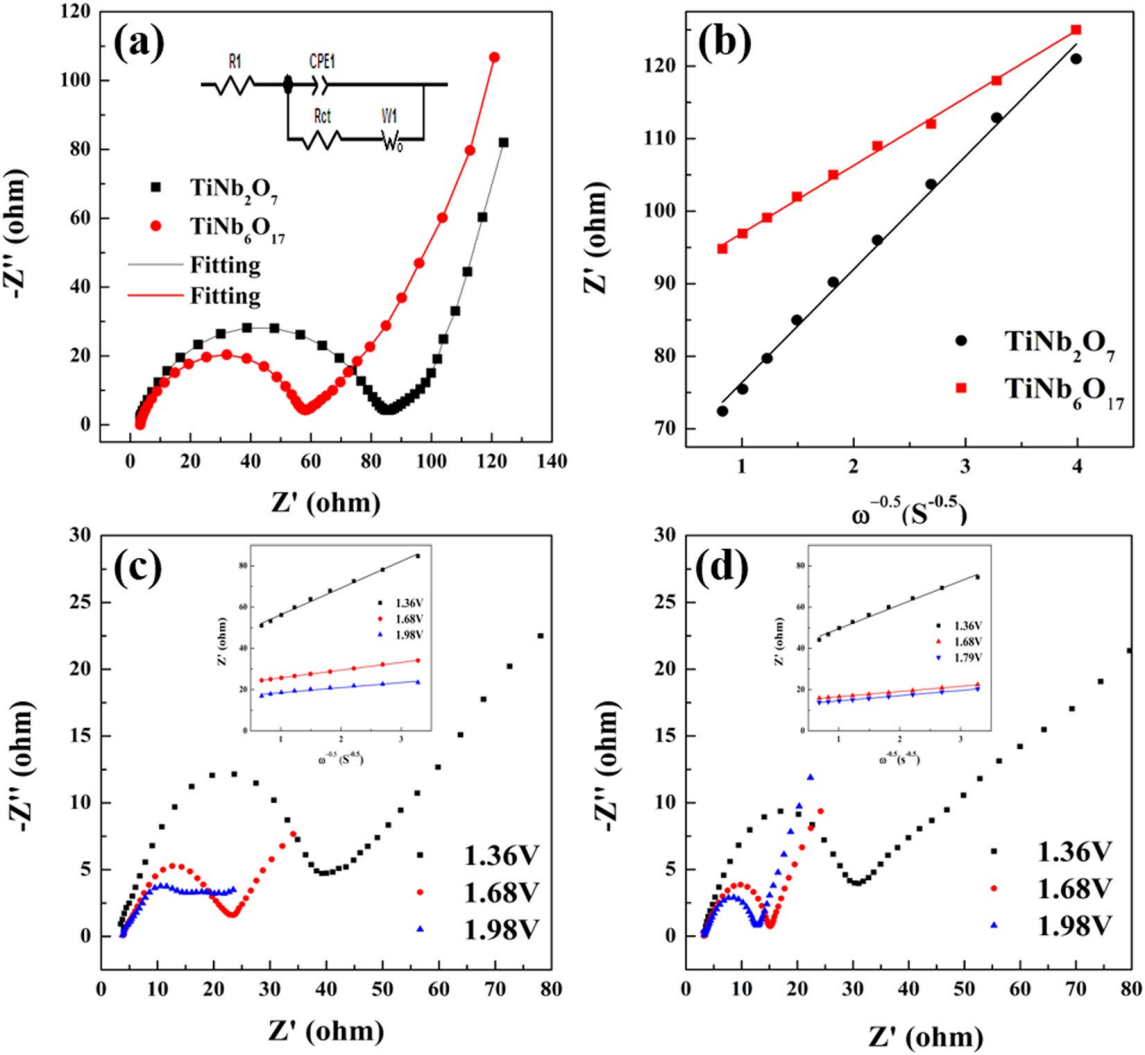
(**a**) Nyquist plots of TiNb_2_O_7_ and TiNb_6_O_17_ anodes at OCV state, (**b**) relationship between imaginary resistance (Z’) and inverse square root of angular speed (at ${\rm{\omega }}$ ^−0.5^) low frequency region, (**c**) Nyquist plots of TiNb_2_O_7_ and (**d**) TiNb_6_O_17_ (Inert images: relationship between Z’ and ${\rm{\omega }}$ ^−0.5^).

Figure 9 presents the Nyquist plots of TiNb_2_O_7_ and TiNb_6_O_17_ by EIS. EIS has been used to examine electrode materials because it can reveal the relationship between the crystal lattice with the electrochemical properties^24–29^. This technique provides kinetic information that can be related to a specific state-of-charge or discharge (SOC, SOD), because the measurement is run by applying a low amplitude signal around an equilibrium state^26–29^. Figure 9(a) shows the Nyquist plot of TiNb_2_O_7_ and TiNb_6_O_17_ at the open circuit voltage (OCV) and an equivalent circuit (insert image). Each Nyquist plot was composed of a high-frequency semicircle and Warburg tail region followed by a steep sloping line in the low-frequency region^27^. The R_1_ and C_dl_ are the ohmic resistance between the electrolyte and surface of the electrode and double layer capacitance. The high-frequency semicircle means the charge-transfer resistance (R_ct_) relevant to the interfacial Li-ion transfer. The Z_w_ is the Warburg impedance, which is related to Li-ion diffusion to the structure of the active materials and corresponds to the tail at a low frequency. Compared to R_ct_, the TiNb_6_O_17_ anode shows a smaller R_ct_ (58Ω) than that of the TiNb_2_O_7_cell (85 Ω). This means that the TiNb_6_O_17_ anode has a faster Li insertion process in the surface area than TiNb_2_O_7_. Figure 9(b) presents a plot of the real part resistance with the inverse square root of the angular speed in the low-frequency range of TiNb_2_O_7_ and TiNb_6_O_17_ anodes at the OCV state. The Warburg factor (${\rm{\sigma }}$) is determined from the slope, and is substituted using equation (Eq. 2 and 3):

2 $$Z^{\prime} ={R}_{{1}}+{R}_{ct}+\sigma {\omega }^{(-1/2)}$$

3 $${D}_{Li}=\frac{{R}^{2}{T}^{2}}{2{A}^{2}{n}^{2}{F}^{4}{C}^{2}{\sigma }^{2}}$$

where Z’ is the real part resistance; $\omega$ is the angular frequency; R is the gas constant; T is the absolute temperature; A is the surface area of the electrode; F is the Faraday constant; and C is the molar concentration of Li ion in an active material. Equations (2) and (3) were used to calculate the Warburg factor and lithium diffusion coefficient, respectively. Table 2 lists the calculated ${{\rm{D}}}_{{{\rm{Li}}}^{+}}$ from the obtained ${\rm{\sigma }}$. The ${{\rm{D}}}_{{{\rm{Li}}}^{+}}$ value of TiNb_2_O_7_ and TiNb_6_O_17_ anodes at the OCV state was 4.57 × 10^–14^ cm^2^s^−1^ and 1.27 × 10^–13^ cm^2^s^−1^, respectively. As a result, the TiNb_6_O_17_ anode has a better lithium diffusion process than the TiNb_2_O_7_ anode. Although it is not the charge/discharge state, the results revealed the improved lithium diffusion kinetics of TiNb_6_O_17_ at the equilibrium state because the more open Li ion insertion site of TiNb_6_O_17_ than that of TiNb_2_O_7_ affects the barrier energy and electrostatic interaction regarding the Li^+^ insertion mechanism.

**Table 2:** Calculated D_LI_^+^ values of (a) TiNb_2_O_7_ and (b) TiNb_6_O_17_ anodes from the EIS results.

	D_Li_^+^ (cm^2^s^−1^) (OCV)	D_Li_^+^ (cm^2^s^−1^) (1.36 V)	D_Li_^+^ (cm^2^s^−1^) (1.68 V)	D_Li_^+^ (cm^2^s^−1^) (1.98 V)
**TiNb**_**2**_ **O**_**7**_	4.57 × 10^−14^	6.64 × 10^−14^	7.91 × 10^−13^	1.85 × 10^−12^
**TiNb**_**6**_ **O**_**17**_	1.27 × 10^−13^	2.94 × 10^−13^	1.12 × 10^−11^	4.57 × 10^−11^

To investigate the Li-ion diffusion properties of two TNO materials at the charge state, *ex-situ* EIS experiments were performed on TiNb_2_O_7_ and TiNb_6_O_17_ anodes at three oxidation reaction potentials of Nb^3+^→ Nb^4+^(1.36 V), Nb^4+^→ Nb^5+^(1.68 V), and Ti^3+^→ Ti^4+^(1.98 V) in Fig. 6 (c),(d). Before the EIS experiments, the discharge and charge were processed during 1 cycle and the discharge was then conducted to the cut off potential of 1.0 V. Figure 9(c) TiNb_2_O_7_ and (d) TiNb_6_O_17_ present Nyquist plots of the two anodes from EIS (Inert images: plot of the real part resistance with the inverse square root of angular speed in the low-frequency range at three oxidation potential). The calculated ${{\rm{D}}}_{{{\rm{Li}}}^{+}}$ value is listed in Table 2 with a value at the OCV state. Compared to ${{\rm{D}}}_{{{\rm{Li}}}^{+}}$ of two TNO anodes from EIS, TiNb_6_O_17_ showed higher ${{\rm{D}}}_{{{\rm{Li}}}^{+}}$ values of 2.94 × 10^−13^ cm^2^s^−1^, 1.12 × 10^−11^ cm^2^s^−1^, and 1.85 × 10^−12^ cm^2^s^−1^ at 1.36 V, 1.68 V, and 1.98 V, respectively, than those of TiNb_2_O_7_ (6.64 × 10^−14^ cm^2^s^−1^, 1.12 × 10^−11^ cm^2^s^−1^, and 4.57 × 10^−11^cm^2^s^−1^). The ${{\rm{D}}}_{{{\rm{Li}}}^{+}}$ values of TiNb_6_O_17_ were 4.4 times (1.36 V), 14 times (1.68 V), and 25 times (1.98 V) higher than those of TiNb_2_O_7_. Compared to the SSCV results, the ${{\rm{D}}}_{{{\rm{Li}}}^{+}}$ values of TiNb_6_O_17_ showed different multiples except for the value at 1.68 V (20, 12, and 38 times at A_p1_-1.36 V, A_p2_-1.68 V, and A_p3_-1.98 V, respectively, from SSCV) but exhibited similar tendency showing higher ${{\rm{D}}}_{{{\rm{Li}}}^{+}}$ at all redox potentials than TiNb_2_O_7_. In particular, the gap of ${{\rm{D}}}_{{{\rm{Li}}}^{+}}$ at 1.68 V (A_p2_ peak at CV) meaning that the two phase regions coincide well with the results of SSCV (14 and 12 fold, respectively.) Therefore, TiNb_6_O_17_ has better lithium diffusion properties than TiNb_2_O_7_ due to its structure inducing a larger open lithium site and a number of Li-ion transport paths during the charge processes.

**Figure 10:**
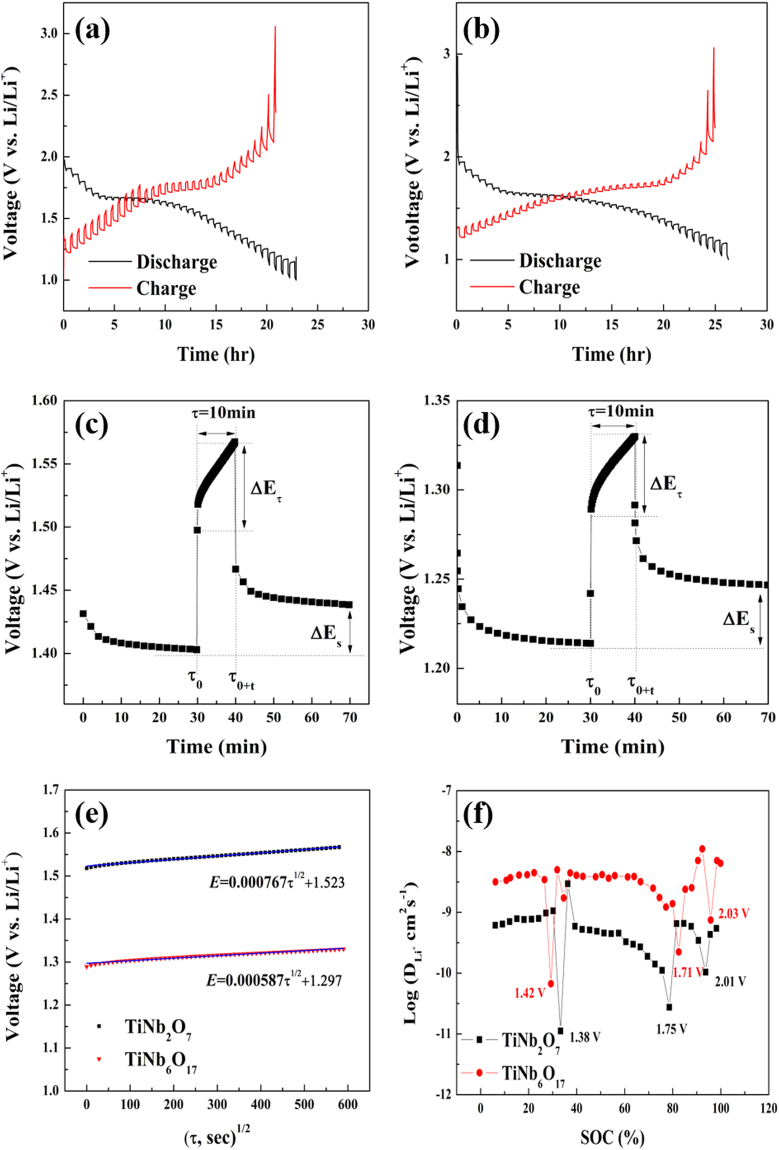
Charge/discharge GITT curves of (**a**) TiNb_2_O_7_ and (**b**) TiNb_6_O_17_, single step of the relationship of single steps for (**c**) and (**d**) (V vs. τ^1/2^), (**e**) and (**f**) lithium diffusion coefficients calculated from GITT for TiNb_2_O_7_ and TiNb_6_O_17_ as a function of the SOC at the charge process.

GITT was conducted to determine the Li^+^ chemical diffusion coefficient and analyze the phase transition of the two TNO materials. The techniques developed by Weppner and Huggins assumed one-dimensional diffusion in a solid solution electrode and a uniform current distribution throughout the electrode and estimated the electrochemically active area from the structure of the active material particles not for the diffusion reaction between the electrode surface and electrolyte^31–37^. At the transitional GITT, a small constant current was applied to an electrode during a short time and the electrode was left to stand after reaching the OCV state^31–35^. In this study, GITT was performed on the TNO materials to determine the ${{\rm{D}}}_{{{Li}}^{+}}$ at a single phase and two phase region as a function of the voltage for the cut off range of the charge/discharge cycle, 1.0–3.0 V. Figure 10 shows the GITT curves of (a) TiNb_2_O_7_ and (b) TiNb_6_O_17_ during the second cycle. The cells were first discharged at a constant current (0.1 C) for a duration time of 15 min and a rest time of 30 min. The curves showed a similar shape and exhibited three plateau regions meaning the solid-solution regions (Ti^4+^ ↔ Ti^5+^ and Nb^3+^ ↔ Nb^4+^) and two-phase reaction (Nb^4+^ ↔ Nb^5+^) with the charge-discharge curves. These regions also showed the cyclic voltammetry peaks of C_p1_ (Ti^4+^ → Ti^3+^), C_p2_ (Nb^5+^ → Nb^4+^), and C_p3_ (Nb^4+^ → Nb^3+^); A_p1_, A_p2_, and A_p3_ mean the oxidation reaction of Nb^3+^ → Nb^4+^, Nb^4+^ → Nb^5+^, and Ti^3+^ → Ti^4+^in Fig. 6(c),(d), Fig. 10 (c) TiNb_2_O_7_ and (d) TiNb_6_O_17_ present the single steps of GITT. The steps are the results measured at the 3^th^ step during the charge state for the same duration and rest time. In Fig. 10(c) and (d), ΔE_τ_ and ΔE_s_ shows the change in the cell voltage during the duration time of 15 min from ${\tau }_{0}$ to ${\tau }_{0+t}$ and the variation of the cell voltage during the rest time of 30 min. The voltage changes from the steps are recorded as a function of time and the lithium diffusion coefficient were calculated using the following equation based on Fick’s second law^32^.

4 $${D}_{L{i}^{+}}=\frac{4}{\pi }{(\frac{{m}_{B}{V}_{M}}{{M}_{B}A})}^{2}{(\frac{{\rm{\Delta }}{E}_{S}}{\tau (d{E}_{\tau }/d\sqrt{\tau }})}^{2}({\rm{\tau }}\ll \frac{{L}^{2}}{{D}_{L{i}^{+}}})$$

where V_M_ is the molar volume of the active material; M_B_ is molecular weight of the materials; m_B_ is the mass of the active materials in an electrode; L is the lithium diffusion distance (thickness of the electrode); A is the electrode area; and $\tau$ is the duration time. When the change in cell voltage with duration time exhibited a linear relationship on plotting against τ^1/2^, equation (4) can be changed to the following simple equation^32^

5 $${D}_{L{i}^{+}}=\frac{4}{\pi \tau }{(\frac{{m}_{B}{V}_{M}}{{M}_{B}A})}^{2}{(\frac{{\rm{\Delta }}{E}_{S}}{{\rm{\Delta }}{E}_{S}})}^{2}({\rm{\tau }}\ll \frac{{L}^{2}}{{D}_{L{i}^{+}}})$$

This equation assumes that the molar volume (V_M_) is stable with the change in Li content in an active material. In this study, the Li^+^ diffusion coefficient of the two TNO materials could be calculated, as shown in Fig. 10. (c)–(e). Figure 10.(e) shows the linear relationship between the single steps in Fig. 10. (c),(d). The Li^+^ diffusion coefficients of the two TNO materials from the GITT results are presented as a function of SOC (%) vs. Log ($${D}_{{{Li}}^{+}})$$ during the charge state in Fig. 10. (f). The coefficients were calculated at all steps during the GITT measurements except for the 1^st^ step and the end two steps of the end due to the large voltage variations. The two cells showed three minimum Li^+^ diffusion coefficient points in Fig. 10.(f) and the voltages representing the points are shown. These minimum diffusion coefficients suggest a phase transition for strong attractive interactions between the intercalation species and the host matrix or some order-disorder transition during cycling^24,32^. Compared to the SSCV and EIS results, the voltages are the three redox potentials of TNO materials, in which the cell voltages of TiNb_2_O_7_ and TiNb_6_O_17_ are 1.42 V~1.38 V (Nb^3+^ → Nb^4+^), 1.71~1.75 V (Nb^4+^ → Nb^5+^), and 2.01~2.03 V (Ti^3+^ → Ti^4+^) vs. 1.36 V, 1.68 V, and 1.98 V, respectively, from the SSCV and EIS measurements. This explains why the plot from GITT has an electrochemical reaction mechanism of two TNO materials with SSCV and EIS. The Li^+^ diffusion coefficients of TiNb_2_O_7_ and TiNb_6_O_17_ from three points were calculated to be 1.11 × 10^−11^ and 6.70 × 10^−11^ cm^2^s^−1^ (Nb^3+^ → Nb^4+^), 2.74 × 10^−11^ and 2.23 × 10^−10^ cm^2^s^−1^ (Nb^4+^ → Nb^5+^), and 1.03 × 10^−10^ and 7.47 × 10^−10^ cm^2^s^−1^ (Ti^3+^ → Ti^4+^). The coefficients of TiNb_6_O_17_ showed higher values than that of TiNb_2_O_7_ at all positions with the other Li^+^ diffusion measurements, which indicates that TiNb_6_O_17_ has superior Li^+^ diffusion kinetics than TiNb_2_O_7_ owing to its larger unit cell volume. Compared to the diffusion coefficients of each transition region, the values increased from the (Nb^3+^ → Nb^4+^) reaction to the (Ti^3+^ → Ti^4+^) reaction, which correspond to the EIS results in Table 2. Among the three diffusion values, the diffusion coefficients of the (Nb^4+^ → Nb^5+^) reaction showed the largest increase from TiNb_2_O_7_ to TiNb_6_O_17_ and also corresponds to the EIS results. These trends suggest that the oxidation reaction is a two phase transition region of TNO materials with the charge-discharge curves and cyclic voltammetry results (the most reaction region). In the event of SSCV, the measurements showed a different tendency with EIS and GITT. The coefficients of the (Nb^4+^ → Nb^5+^) reaction (A_p2_) showed the largest values and the diffusion coefficients of the (Nb^3+^ → Nb^4+^) reaction showed the largest increase from TiNb_2_O_7_ to TiNb_6_O_17._ This may be due to the inaccuracy of the SSCV measurements in this study. Compared to A_p2_, both A_p1_ and A_p3_ showed a small peak current and a broad shape. Therefore, the diffusion coefficients of the two peaks may be not precise values.

## Conclusions

Galvanostatic charge-discharge, cyclic voltammetry, and rate capability test were conducted to analyze the electrochemical performance and properties of TiNb_6_O_17_ and TiNb_2_O_7_. From the results, two TNO materials showed three similar plateau regions and three redox peaks corresponding to two Nb redox and one Ti redox reaction. TiNb_6_O_17_ showed higher capacities of 284mAh/g than that of TiNb_2_O_7_ 264mAh/g. In the rate capability test, TiNb_6_O_17_ exhibited improved rate capacity of 80mAh/g at 30 C than 19mAh/g for TiNb_2_O_7_. SSCV, EIS, and GITT measurement were taken to investigate the performance and lithium diffusion properties related to the unit cell volume of the two TNO materials. The anodic and cathodic Li^+^ diffusion coefficients from SSCV were in the range of 10^−14^ to 10^−15^ cm^2^s^−1^ for TiNb_2_O_7_ and 10^−13^ to 10^−14^ cm^2^s^−1^ for TiNb_6_O_17_. The anodic diffusion coefficients of TiNb_6_O_17_ were 5 times (Nb^3+^ → Nb^4+^), 15 times (Nb^4+^ → Nb^5+^), and 14 times (Ti^3+^ → Ti^4+^). From the EIS measurement, the coefficients were in the range of 10^−12^ to 10^−14^cm^2^s^−1^ of TiNb_2_O_7_ and 10^−11^ to 10^−13^cm^2^s^−1^ of TiNb_6_O_17_ at the OCV state and three oxidation potential region of the two TNO materials during the charging process. The three minimum diffusion coefficients points were determined from the GITT measurement. The diffusion coefficients of the two phase transition region (Nb^4+^ → Nb^5+^) were improved 10 fold compared to that of TiNb_2_O_7_. CV, EIS, and GITT indicated that TiNb_6_O_17_ has better lithium diffusion kinetics and electrochemical performance than TiNb_2_O_7_ because of its large unit cell volume and more open Li^+^ insertion site.
